# Recent Perspectives on the Mechanism of Recurrence After Ablation of Hepatocellular Carcinoma: A Mini-Review

**DOI:** 10.3389/fonc.2022.895678

**Published:** 2022-08-23

**Authors:** Jianquan Yang, Wen Guo, Man Lu

**Affiliations:** ^1^ The School of Medicine, University of Electronic Science and Technology of China, Chengdu, China; ^2^ Department of Ultrasound Medical Center, Sichuan Cancer Hospital and Institute, Sichuan Cancer Center, School of Medicine, University of Electronic Science and Technology of China, Chengdu, China; ^3^ Institute of Materia Medica, North Sichuan Medical College, Nanchong, China

**Keywords:** hepatocellular carcinoma, early stage, ablation, recurrence, mechanism

## Abstract

Hepatocellular carcinoma (HCC) is one of the most common malignant tumors. Hepatectomy, liver transplantation, and ablation are the three radical treatments for early-stage hepatocellular carcinoma (ESHCC), but not all patients are fit for or can tolerate surgery; moreover, liver donors are limited. Therefore, ablation plays an important role in the treatment of ESHCC. However, some studies have shown that ablation has a higher local recurrence (LR) rate than hepatectomy and liver transplantation. The specific mechanism is unknown. The latest perspectives on the mechanism of recurrence after ablation of HCC were described and summarized. In this review, we discussed the possible mechanisms of recurrence after ablation of HCC, including epithelial–mesenchymal transition (EMT), activating autophagy, changes in non-coding RNA, and changes in the tumor microenvironment. A systematic and comprehensive understanding of the mechanism will contribute to the research and development of related treatment, combined with ablation to improve the therapeutic effect in patients with ESHCC.

## 1 Introduction

HCC is a common malignant tumor in China, with the fourth-highest morbidity and the second-highest mortality rate (1, 2); thus, early diagnosis and treatment are important to improve survival, especially for patients with early-stage hepatocellular carcinoma (ESHCC). The main treatments for ESHCC are liver transplantation, hepatectomy, and ablation. Liver transplantation is one of the radical treatments, but there are some limitations such as the limited liver donors and postoperative immune rejection (3). Child-Pugh A grade is required for liver function in HCC patients receiving hepatectomy. Most ESHCC patients have a history of hepatitis B or liver cirrhosis, and the liver reserve is poor, these patients are at high risk for hepatectomy (4). Ablation, as one of the three radical treatments for ESHCC, has the advantage of being minimally invasive and having limited impact on liver function; thus, inoperable ESHCC patients may choose radical treatment with ablation (5–7). Moreover, there was no difference in overall survival (OS) time between ablation and hepatectomy in ESHCC patients (8). However, some studies have shown that ablation has a higher local recurrence (LR) rate than hepatectomy (9, 10). The mechanism is still unclear. In recent years, many studies have explored the possible mechanism behind the higher LR rate in ESHCC after ablation. In this paper, recent perspectives were reviewed.

## 2 Commonly Used Local Ablation Treatment

### 2.1 Thermal Ablation

#### 2.1.1 Radiofrequency Ablation

Radiofrequency ablation (RFA) uses radiofrequency alternating current to generate heat between the percutaneous probe and the surrounding tissues, resulting in an increase in temperature between 60°C and 100°C, which is maintained for several minutes, leading to complete coagulative necrosis in the tumor and surrounding tissues (11, 12). RFA has become the main local ablation method for the treatment of ESHCC due to its advantages (it is safer, has less trauma, is less painful, has better tolerance, has fewer adverse reactions, and has quicker recovery) (13). However, the scope of coagulation necrosis caused by RFA is limited; the complete ablation rate of ESHCC with a diameter of more than 3 cm is significantly decreased; and the rate of LR is higher than surgical resection (14). Some studies have reported that RFA combined with transcatheter arterial chemoembolization (TACE) or other treatments could increase the ablation scope and therapeutic efficacy (15). In addition, several studies have reported that RFA combined with chemotherapy drugs or targeted drugs such as sorafenib can improve the therapeutic effect in HCC and prolong the survival time of patients (16). However, the thermal deposition effect [heat-sink effect (HSE); that is, the temperature of the ablation zone is decreased when tumors are located near blood vessels or bronchia] reduces the efficacy of RFA. Therefore, when the tumor is near blood vessels or bronchia (17, 18), the complete ablation rate of RFA will decrease (19). At the same time, RFA may cause skin cauterization, which is one of the major side effects of RFA (20).

#### 2.1.2 Microwave Ablation

Microwave ablation (MWA), another type of thermal ablation, aims to generate an electromagnetic field of 900–2,500 MHz through an antenna placed in the tumor. The electromagnetic field continuously realigns the polar molecules with inherent dipoles in the tissue with the oscillating electric field, and the rotation of the molecules increases their kinetic energy, thus increasing the temperature in the tissues (21, 22). As early as 2001, MWA has been used in the treatment of ESHCC, and several studies have confirmed that MWA has long-term efficacy and could effectively reduce adverse reactions (23, 24). With the continuous progress of MWA technology, MWA has been used from the initial treatment of small HCC to the combination with TACE in the treatment of large HCC and HCC in special areas, such as tumors near the gallbladder, gastrointestinal tract, large vessels, and hilum (24–27). MWA is an important treatment for ESHCC. Liang et al. have led many innovative studies on the treatment of ESHCC with MWA (28, 29) and have formulated the first guidelines on treating ESHCC *via* MWA (30), laying a theoretical foundation for clinicians to standardize the operation. Compared with RFA, MWA has a lower HSE rate (31, 32) and may result in fewer instances of skin burn (33). Most importantly, MWA has a larger ablation scope than RFA (34, 35), and research has shown that for HCCs that are 5–7 cm in diameter, the complete ablation rate of MWA is 80%, while that of RFA is only 24% (36).

### 2.2 Other Ablation Treatments

#### 2.2.1 Cryoablation

The goal of cryoablation (CA) is to form ice in the cells and maintain the temperature for a few minutes/cycles, eventually leading to cell death, with a lethal temperature range of −40 °C to −20 °C (37). Compared with RFA and MWA, CA has a lower incidence of pain (38). Due to the different theory of CA, an ice hockey ablation area (IBAZ) can be formed during ablation, which enables CA to monitor the scope and size of the ablation area in real time through imaging devices such as computed tomography (CT), magnetic resonance imaging (MRI), or ultrasound (39–41). However, about 1% of CA may induce hypothermic shock, some of which are fatal (42). In addition, the long-term efficacy of CA in HCC needs more follow-up data (43).

#### 2.2.2 Irreversible Electroporation

Irreversible electroporation (IRE) destroys the tissues and cell membranes by generating a non-thermal electric field on the ablation probe, thus changing the ion-driven homeostasis, resulting in cell death (44). The greatest advantage of IRE is that it can protect the bile ducts and blood vessels around the ablation area from ablation damage (45). Because IRE has a broad transition zone between surviving tissue and necrotic tissue, it may induce LR (46). Meanwhile, as a new form of ablation, the data on IRE’s applicability to other types of diseases and long-term efficacy are limited; thus, it needs more research to verify its therapeutic effect (47, 48).

Although there are many methods of local ablation, RFA and MWA, two forms of thermal ablation, are mainly used for ESHCC in the clinical setting. Next, we will discuss and summarize the mechanism of HCC recurrence caused by thermal ablation.

## 3 The Mechanism of Recurrence After Ablation of HCC

### 3.1 EMT

Epithelial–mesenchymal transition (EMT) refers to the transformation from epithelial cells to mesenchymal cells, the molecules of which are remodeled, and phenotypic changes, including malignant tumor progression, enhance the ability of invasion and metastasis in epithelial-derived tumor cells (49). Molecular marker changes in EMT include losing the expression of epithelial markers such as E-cadherin, upregulation of mesenchymal cell markers such as N-cadherin and Vimentin, and the increasing transcriptional regulatory factors such as Snail, Slug, and Twist (50). To investigate whether EMT occurs in recurrence after ablation in HCC, Yoshida et al. simulated thermal ablation *in vitro* by sublethal heat stress (SHS); that is, hepatoma cells were placed in a water bath at 45°C and 55°C for 10 min, the morphology of the surviving tumor cells was spindle-like on the 5th day, and the expression of CD133, CK7, CK19, and Snail proteins increased, but returned to the baseline level on the 12th day (51). It showed that the surviving hepatoma cells after SHS are capable of EMT, and further research showed that SHS activated the Erk1/2 signal pathway; inhibiting the Erk1/2 pathway could inhibit the proliferation of hepatoma cells. Dong et al. found that SHS-treated hepatoma cells increased the expression of PCNA, N-cadherin, MMP-2, and MMP-9 through activating the Akt/ERK pathway. Furthermore, an animal experiment showed that the risk of lung metastasis was increased with insufficient ablation (52). One research, through an analysis of specimens from recurrent HCC in patients who have undergone ablation, showed that EMT-related markers TGF-β, Twist, and Snail-1 increased compared with patients without ablation. In order to further confirm the role of EMT in recurrence after thermal ablation of HCC, some researchers constructed an *in situ* incomplete HCC ablation in a xenograft mouse model and found that E-cadherin was downregulated while N-cadherin and β-catenin were upregulated, and that blocking β-catenin, a key protein of EMT, could reduce the EMT phenotype and metastasis in hepatoma cells (53). As a key protein of EMT, flotillin can activate the Akt/Wnt/β-catenin signal pathway to induce EMT and then enhance the ability of proliferation and invasion in residual hepatoma cells after SHS (54). As a key step to obtaining stronger invasion and metastasis, EMT has been confirmed in hepatoma cells, animal models, and samples from HCC patients, and some studies find some key proteins and important signal pathways leading to EMT (**Table 1**), which provide the direction for basic research and clinical treatment in the future.

**Table 1:** EMT in recurrence after ablation of HCC.

Researchers	Experimental Methods	Research Results	Ref.
Yoshida et al.	*In vitro*	The expression of CD133, CK7, CK19, and Snail proteins increased.	(51)
Dong et al.	*In vitro*	The expression of PCNA, N-cadherin, MMP-2, and MMP-9 increased.	(52)
Zhang et al.	*In vivo* and patient’s surgical sample	EMT-related markers TGF-β, Twist, and Snail-1 increased.	(53)
Zhang et al.	*In vitro*	Fettillins activated Akt/Wnt/β-catenin pathway to induce EMT.	(54)

### 3.2 Promoting Angiogenesis Through the HIF-1α/VEGF Signal Pathway

As an important regulator of hypoxic adaptive response, HIF-1α is highly expressed in hypoxic conditions but is maintained at a low concentration under normoxic conditions (55, 56). HIF-1α is usually more obvious in invasive tumors (56) and can be used as an independent predictor for poor prognosis in HCC (57). HIF-1α plays an important role in promoting proliferation, invasion, angiogenesis, and metastasis in tumors (58). Angiogenesis plays a key role in tumor formation and metastasis (59, 60). HIF-1α directly induces a large number of angiogenesis-related genes, such as the VEGF family. The VEGF family, including VEGFA, VEGFB, VEGFC, and VEGFD, are the strongest angiogenic factors expressed in a variety of tumors (61). Studies have shown that VEGF is highly expressed in HCC (62). The expression of VEGF in HCC tissues or serum implies the vascular invasion and metastasis of HCC, which leads to an increase in the recurrence of HCC after treatment and a decrease in the median survival time of patients (63–66). VEGF is the main regulatory factor of angiogenesis. Previous research has shown that VEGF is highly expressed in tumors, and anti-angiogenesis therapy with VEGF inhibitors can inhibit tumor growth (67). Since bevacizumab (Avastin) has been approved in 2004, Avastin has been shown to be effective against a variety of solid tumors, including HCC (68). After the breakthrough of immunotherapy in some solid tumors, studies have shown that immunotherapy plus anti-angiogenesis can further improve efficacy in HCC patients, and has become the first-line treatment for advanced HCC (69). Sorafenib, another anti-angiogenic drug, is used for advanced HCC, which could prolong survival time in patients with advanced HCC (70). A series of retrospective studies have shown that RFA combined with sorafenib can reduce the postoperative LR rate and improve the survival time of patients with HCC (71, 72). However, a randomized, placebo-controlled, double-blind Phase 3 trial showed that there was no significant difference in relapse-free survival time between sorafenib with RFA and RFA with placebo (73). Compared with parent cells, hepatoma cells treated with SHS increase the expression of HIF-1α and VEGF, and enhance angiogenesis, which can be eliminated by Avastin (74). Liu et al. found that hepatoma cells treated with SHS promoted proliferation by the overexpression of CaMKII/ERK-dependent VEGF. Similarly, in the ectopic liver cancer model, it also showed that HIF-1α, VEGF, and microvessel density (MVD) increased after insufficient ablation (75). Animal models confirm that incomplete ablation promotes angiogenesis through the HIF-1α/VEGF signal pathway leading to tumor invasion and metastasis (76). Tan et al. found that incomplete ablation led to the high expression of VEGFR1 and enhanced angiogenesis, and that targeting VEGFR1 could reduce the ability of proliferation and migration in hepatoma cells (77). Analysis of the samples of patients with recurrent HCC after ablation showed that the level of HIF-1α was higher than without RFA. Survival analysis showed that OS was shorter in patients with recurrence after RFA (78). A series of studies have revealed that promoting angiogenesis enrolls in the recurrence of HCC after ablation by activating the HIF-1α/VEGF signal pathway (**Table 2**). Anti-angiogenesis through targeted HIF-1α / VEGF pathway may be one of the ways to treat recurrence of HCC after ablation, but its clinical use needs more basic researches and clinical trials.

**Table 2:** Promoting angiogenesis in recurrence after ablation of HCC.

Researchers	Experimental Methods	Research Results	Ref.
Kong et al.	*In vitro*	The expression of HIF-1 α and VEGF increased in hepatoma cells treated with SHS.	(74)
Liu et al.	*In vitro*	VEGF was overexpressed in hepatoma cells treated with SHS.	(75)
Wu et al.	*In vivo*	Incomplete ablation promoted angiogenesis through HIF-1α/VEGF signal pathway leading to tumor invasion and metastasis.	(76)
Tan et al.	*In vivo*	Incomplete ablation led to high expression of VEGFR1 and enhanced angiogenesis.	(77)
Yamada et al.	Patient’s surgical sample	The level of HIF-1α was higher in patients with recurrent HCC after ablation.	(78)

### 3.3 Activating Autophagy

Autophagy is a self-protective mechanism in cells, which can degrade cytoplasmic substances such as damaged organelles and proteins (79, 80). Recently, there is considerable evidence showing that autophagy plays a key role in many human diseases, including tumors. Inhibiting autophagy can prevent tumor progression (81). The level of LC3-II (a key autophagy marker) is positively correlated with poor prognosis in HCC (82). Chang et al. found that inhibition of autophagy reduced the survival of hepatoma cells (83). In addition, autophagy can help hepatoma cells survive under adverse conditions and promote invasion and migration (84). Peng et al. confirmed that autophagy induced by hypoxia led to resistance to chemotherapeutic drugs in hepatoma cells (85). Thompson et al. found that SHS-treated hepatoma cells activated the PI3K/mTOR/AKT pathway, an important signal pathway of autophagy, resulting in enhanced proliferation and invasion (86). Jondal et al. also showed that the ability of invasion was enhanced in hepatoma cells by SHS by activating the PI3K/mTOR/AKT pathway, and inhibition of the PI3K/mTOR pathway was an effective approach against invasion (87). The autophagy inhibitor chloroquine (CQ) can inhibit the growth of residual hepatoma cells after SHS treatment (88). Zhao et al. confirmed the role of autophagy in an ectopic animal model for the first time by incomplete ablation [insufficient RFA (IRFA)], which promoted proliferation and invasion in residual hepatoma cells. The autophagy inhibitor hydroxychloroquine (HCQ) can also significantly inhibit the growth of hepatoma cells (89). Autophagy is an important mechanism in the tumorigenesis and progression of malignant tumors; activating autophagy also exists in the recurrence of HCC after ablation (**Table 3**). *In vitro* and *in vivo*, it has been shown that autophagy inhibition can inhibit the progression of HCC after ablation.

**Table 3:** Activating autophagy in recurrence after ablation of HCC.

Researchers	Experimental Methods	Research Results	Ref.
Thompson al.	*In vitro*	The PI3K/mTOR/AKT signal pathway of autophagy was activated by SHS-treated hepatoma cells.	(86)
Jondal et al.	*In vitro*	The PI3K/mTOR/AKT signal pathway of autophagy was activated by SHS-treated hepatoma cells.	(87)
Jiang et al.	*In vitro*	CQ can inhibit the growth of residual hepatoma cells after SHS treatment.	(88)
Zhao et al.	*In vitro*	HCQ can significantly inhibit hepatoma cell growth.	(89)

### 3.4 Changes in Non-Coding RNA

**Figure 1:**
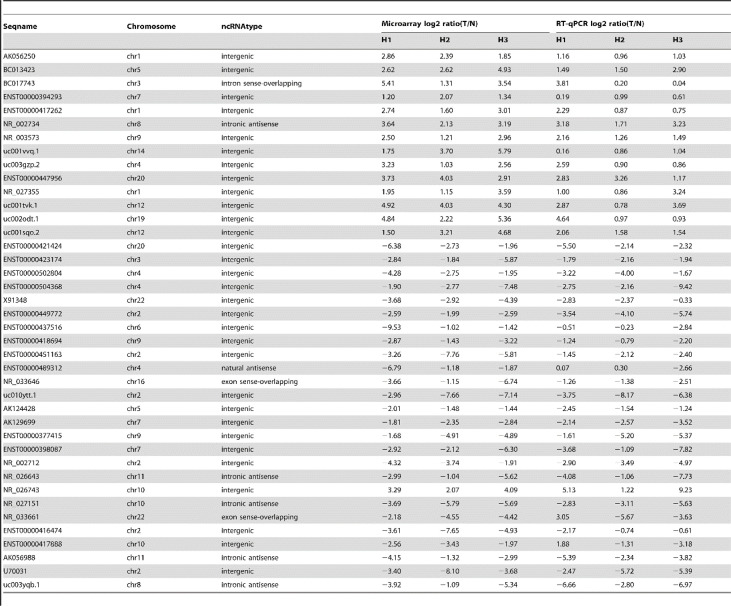
Quantitative RT-PCR confirmation for forty selected lncRNA, cited from Zhu et al. *Plos One.* 2014 Jul 15;9(7): e101707 (91). .

Long-stranded non-coding RNA (lncRNA) is usually defined as a transcript with more than 200 nucleotides lacking the function of protein coding. In recent years, nevertheless, lncRNA has shown an important regulatory role in cancer biology, including immune response, tumorigenesis, cell development, and metabolism (90, 91). The expression of lncRNA was detected by a gene chip, and hundreds of lncRNA were abnormally expressed in HCC (**Figure 1**) (91). It was found that the expression of lncRNA and mRNA in hepatoma cells treated with SHS was significantly changed; about 558 lncRNA and 250 mRNA were upregulated, while 224 lncRNA and 1,031 mRNA were downregulated. Further studies showed that the expression of lncRNA FUNDC2P4 in SHS-treated hepatoma cells promoted EMT by reducing the expression of E-cadherin, which promoted proliferation and invasion in hepatoma cells. The downregulation of miR-200C and miR-34a expression promoted EMT and enhanced the invasiveness of SHS-treated hepatoma cells (91). As a current research hotspot, there are some studies that focused on the change of non-coding RNA in the recurrence of HCC after ablation, and more studies are needed to reveal its particular role in the recurrence of HCC after ablation.

### 3.5 Changes in the Tumor Microenvironment

The mechanisms described above are all based on hepatoma cells. However, the tumor microenvironment (TME) also plays an important role in tumorigenesis and tumor progression. TME is rich in activated hepatic stellate cells (HSCs). Evidence shows that the activated HSCs promote hepatoma cells’ proliferation, migration, and invasion (92–95). Periostin (POSTN) is a key protein that activates HSCs and is a perfect anti-fibrosis target (96). The high expression of POSTN can induce EMT and promote proliferation in hepatoma cells (97) and can lead to a worse prognosis in patients with HCC (98). Zhang et al. exposed hepatoma cells to SHS and then cultured them with activated hepatic stellate cell conditioned medium (HSC-CM). Subsequently, there was enhanced invasion of residual hepatoma cells through HSC-CM secreting POSTN. It is proved that POSTN plays a vital role in the recurrence of HCC after ablation (99). It is further found that POSTN induced residual hepatoma cells by SHS, acquiring stem cell characteristics through activating the integrin β-1/AKT/GSK3-β/β-catenin/TCF4/Nanog pathway (100). Zhang et al. also found that the matrix hardness and extracellular matrix type I collagen were involved in the malignant progression in residual hepatoma cells after SHS (101). ERK1/2 inhibitors can reverse ERK phosphorylation induced by extracellular matrix type I collagen (102). TME plays an important role in tumorigenesis and progression, the researches have conducted a preliminary study on the role of TME in recurrence of HCC after ablation (**Table 4**), but more in-depth studies are needed to find vital proteins and signal pathways.

**Table 4:** TME changes in recurrence after ablation of HCC.

Researchers	Experimental Methods	Research Results	Ref.
Zhang al.	*In vitro*	POSTN played an important role in the recurrence of HCC after ablation.	(99)
Zhang et al.	*In vitro*	POSTN upregulated the stem cell characteristics in residual hepatoma cells by SHS.	(100)
Jiang et al.	*In vitro*	The matrix hardness and extracellular matrix type I collagen increased in residual hepatoma cells *via* SHS.	(101)

## 4 Conclusions

**Figure 2:**
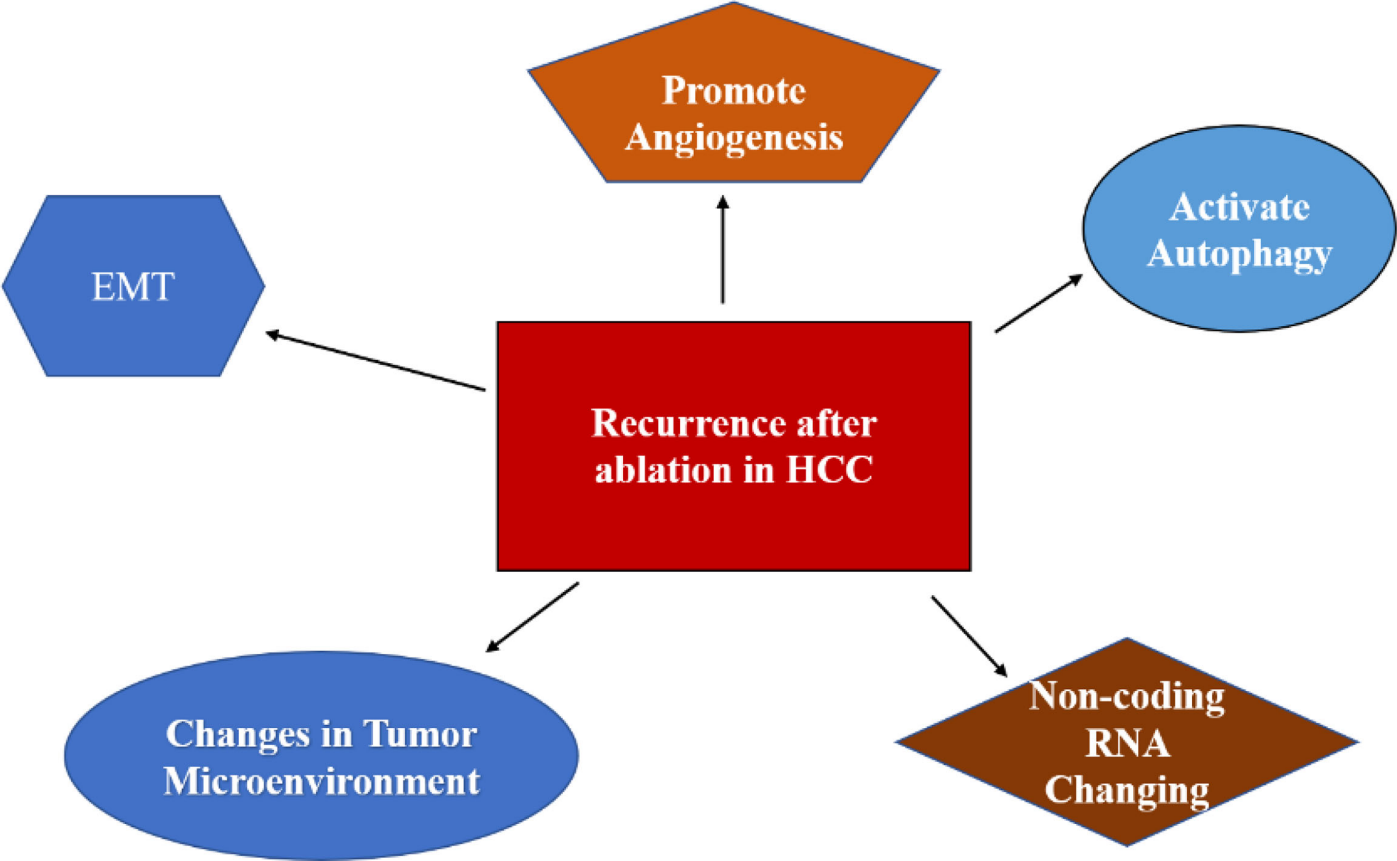
Schematic representation of the mechanism of recurrence after ablation of HCC. .

As a minimally invasive, safe, and effective treatment, ablation plays a major role in ESHCC. The mechanism of recurrence of HCC after ablation is mainly focused on EMT, angiogenesis, autophagy activation, and changes in the TME (**Figure 2**). Current perspectives do not fully elucidate the mechanism of recurrence of HCC after ablation. Most of the studies are *in vivo*, and some are *in vitro*. For the establishment of an incomplete tumor ablation model *in vitro*, most studies used simulated thermal ablation *in vitro* by SHS. Some scholars doubted whether this method could completely mimic the tumor environment. Furthermore, the temperature and time of the water bath are not the same in different experiments, thus lacking uniform standards (49). In the future, we hope to improve the research method *in vitro* and *in vivo*, revealing its distinct mechanism, and providing the theoretical basis for clinical transformation.

## Author Contributions

JQY and WG performed the data collection and analysis. JQY, WG, and ML were responsible for the data interpretation. All authors read and approved the final manuscript.

## Funding

This study was supported by funds from the National Key Research and Development Program (No. 2019YFE0196700).

## Conflict of Interest

The authors declare that the research was conducted in the absence of any commercial or financial relationships that could be construed as a potential conflict of interest.

## Publisher’s Note

All claims expressed in this article are solely those of the authors and do not necessarily represent those of their affiliated organizations, or those of the publisher, the editors and the reviewers. Any product that may be evaluated in this article, or claim that may be made by its manufacturer, is not guaranteed or endorsed by the publisher.

## Abbreviations

HCC, hepatocellular carcinoma; OS, overall survival; LR, local recurrence; RFA, radiofrequency ablation; HSE, heat-sink effect; MWA, microwave ablation; CA, cryoablation; IBAZ, ice ball ablation zone; IRE, irreversible electroporation; EMT, epithelial–mesenchymal transition; SHS, sublethal heat stress; MVD, microvessel density; CQ, chloroquine; IRFA, insufficient RFA; HCQ, hydroxychloroquine; lncRNA, long non-coding RNA; HSCs, hepatic stellate cells; POSTN, periostin; HSC-CM, hepatic stellate cell conditioned medium.
